# Influence of joint angular velocity on electrically evoked concentric force potentiation induced by stretch-shortening cycle in young adults

**DOI:** 10.1186/s40064-015-0875-0

**Published:** 2015-02-13

**Authors:** Atsuki Fukutani, Toshiyuki Kurihara, Tadao Isaka

**Affiliations:** Research Organization of Science and Technology, Ritsumeikan University, 1-1-1 Noji-higashi, Kusatsu, Shiga 525-8577 Japan; Japan Society for the Promotion of Science, 5-3-1 kojimachi, Chiyoda-ku, Tokyo 102-8472 Japan; Faculty of Sport and Health Science, Ritsumeikan University, 1-1-1 Noji-higashi, Kusatsu, Shiga 525-8577 Japan

**Keywords:** Preactivation, Residual force enhancement, Tendon elongation, Fascicle length, Pennation angle

## Abstract

**Background:**

During a stretch- shortening cycle (SSC), muscle force attained during concentric contractions (shortening phase) is potentiated by the preceding eccentric contractions (lengthening phase). The purpose of this study was to examine the influence of joint angular velocity on force potentiation induced by SSC (SSC effect).

**Findings:**

Twelve healthy men (age, 24.2 ± 3.2 years; height, 1.73 ± 0.05 m; body mass, 68.1 ± 11.0 kg) participated in this study. Ankle joint angle was passively moved by a dynamometer, with range of motion from dorsiflexion (DF) 15° to plantarflexion (PF) 15°. Muscle contractions were evoked by tetanic electrical stimulation. Joint angular velocity of concentric contraction was set at 30°/s and 150°/s. Magnitude of SSC effect was calculated as the ratio of joint torque obtained by concentric contraction with preliminary eccentric contraction trial relative to that obtained by concentric contraction without preliminary eccentric contraction trial. As a result, magnitude of SSC effect calculated at three joint angles was significantly larger in the 150°/s condition than in the 30°/s condition (*p* < 0.05).

**Conclusions:**

These results indicate that the magnitude of SSC effect is affected by joint angular velocity, which is larger when joint angular velocity is larger. This phenomenon would be caused by insufficient duration to increase activation level in the large joint angular velocity condition. When the duration to increase activation level is insufficient due to short contraction duration, preactivation (one of the factors of SSC effect) leads to a significant increase in joint torque.

## Findings

### Introduction

It is well known that muscle force production during a concentric contraction is potentiated by conducting eccentric contraction before concentric contraction. This phenomenon is called a stretch-shortening cycle (SSC) (Cavagna et al. 1968; Komi 2000). Many studies have examined force potentiation induced by SSC (SSC effect) using animal (Ettema et al. 1990), human (Finni et al. 2001, 2003), and simulation (Nagano et al. 2004; Arakawa et al. 2010) models.

A previous study reported that the SSC effect was related to joint angular velocity (Svantesson et al. 1994), which was larger in faster velocity condition. However, since the authors used a methodology of voluntary contractions and reported that neural activity was not constant between different joint angular velocities and between use or nonuse of preliminary eccentric contraction, interpretation of the relationship between joint angular velocity and SSC effect is complicated. In this regard, electrically-evoked contractions are a useful method to control muscle activation independently of voluntary effort among contractions. Therefore, the purpose of this study was to re-examine the influence of joint angular velocity on SSC effect using electrically-evoked contractions.

### Methods

#### Participants

Twelve healthy young men (age, 24.2 ± 3.2 years; height, 1.73 ± 0.05 m; body mass, 68.1 ± 11.0 kg) volunteered to participate in the present study. These subjects were recruited from the university population and were recreationally active. The purpose and risks of this study were explained to each volunteer, and written informed consent was obtained. The Ethics Committee on Human Research of Ritsumeikan University approved this study (IRB-2013-14).

#### Settings of joint motion and electrical stimulation

In this study, SSC effect was calculated from plantarflexion (PF) using a dynamometer (Biodex; SAKAImed, Tokyo, Japan) in an isokinetic 30°/s and 150°/s conditions. Attachment of dynamometer (i.e., ankle joint angle) was cyclically-moved with the range of motion from dorsiflexion (DF) 15° to PF15°. The ankle joint angle at the anatomical position (neutral position) was defined as PF0°. The knee and hip joints were fixed at flexed at 0° and 80°, respectively.

To standardize muscle activation among contractions, all contractions were evoked by electrical stimulation (SEN-3401; Nihon Kohden, Tokyo, Japan). Muscle contractions were evoked by muscle belly stimulation. An anode (4 × 5 cm) was placed on the proximal aspect of the triceps surae, while a cathode (4 × 5 cm) was placed on the distal aspect of the soleus. The parameters of electrical stimulation were as follows: pulse frequency, 100 Hz; pulse duration, 0.5 ms; and train duration, 1.5 s. The voltage of the electrical stimulation was corresponding to the value which evoked the 25% intensity relative to the maximal voluntary isometric torque at PF0°. To determine the voltage of electrical stimulation, maximal voluntary isometric contractions were performed with the ankle joint angle at PF0°. The peak joint torque recorded in these contractions was used to determine the voltage of electrical stimulation which was adjusted to evoke 25% intensity at the identical joint angle. This electrical stimulation voltage was applied to all contractions.

To calculate SSC effect in 30°/s and 150°/s conditions, two types of trial were conducted in both velocity conditions. In the control trial, the ankle was rotated passively only in the shortening phase and electrical stimulation was applied at the instance when the ankle joint angle passed DF10° in the shortening phase (i.e., in the phase from DF15° to PF15°) to evoke concentric contraction without preliminary eccentric contraction. Onset of the electrical stimulation was controlled by an AD converter (Power lab 16/30; ADInstruments, Bella Vista, Australia). Specifically, when the signal of joint angle reached the value corresponding to the DF10°, output signal to the electrical stimulation machine was generated by the AD converter. Joint angular velocity of concentric contraction was 30°/s and 150°/s, while that of eccentric contraction was identical (60°/s) in both conditions. On the other hand, in the SSC trial, the ankle joint was first rotated eccentrically and then electrical stimulation was applied at the instance when the ankle joint angle passed PF10° in the lengthening phase (i.e., in the phase from PF15° to DF15°). Immediately after the lengthening, the shortening (i.e., concentric contraction) was conducted. Settings of joint angular velocity were identical to those of the control trial. Joint torque and joint angle were recorded with a sampling frequency of 4,000 Hz (Power lab 16/30; ADInstruments, Bella Vista, Australia).

#### Analyses and measurements

Joint torques recorded at the instances of DF5°, PF0°, and PF5° were used in the following analyses. Joint torques recorded at these three joint angles in the SSC trial were expressed as relative values with respect to those in the control trial. This value was defined as the magnitude of SSC effect. Trials were randomized. In addition, to reduce the influence of random error, all trials were conducted twice, and mean values were adopted for the following analyses in the current study.

#### Statistical analysis

Two-way analysis of variance (ANOVA) with repeated measures was adopted to examine the interaction (velocity × joint angle) and main effect of SSC effect. When the interaction was significant, additional analyses using one-way ANOVA with repeated measures followed by post hoc test (Bonferroni’s correction) for among joint angles, and post hoc test (paired *t*-test) for between velocities were performed. Statistical analyses were performed using SPSS version 20 software (IBM, Tokyo, Japan), with the level of statistical significance set at *p* < 0.05.

### Results

**Figure 1:**
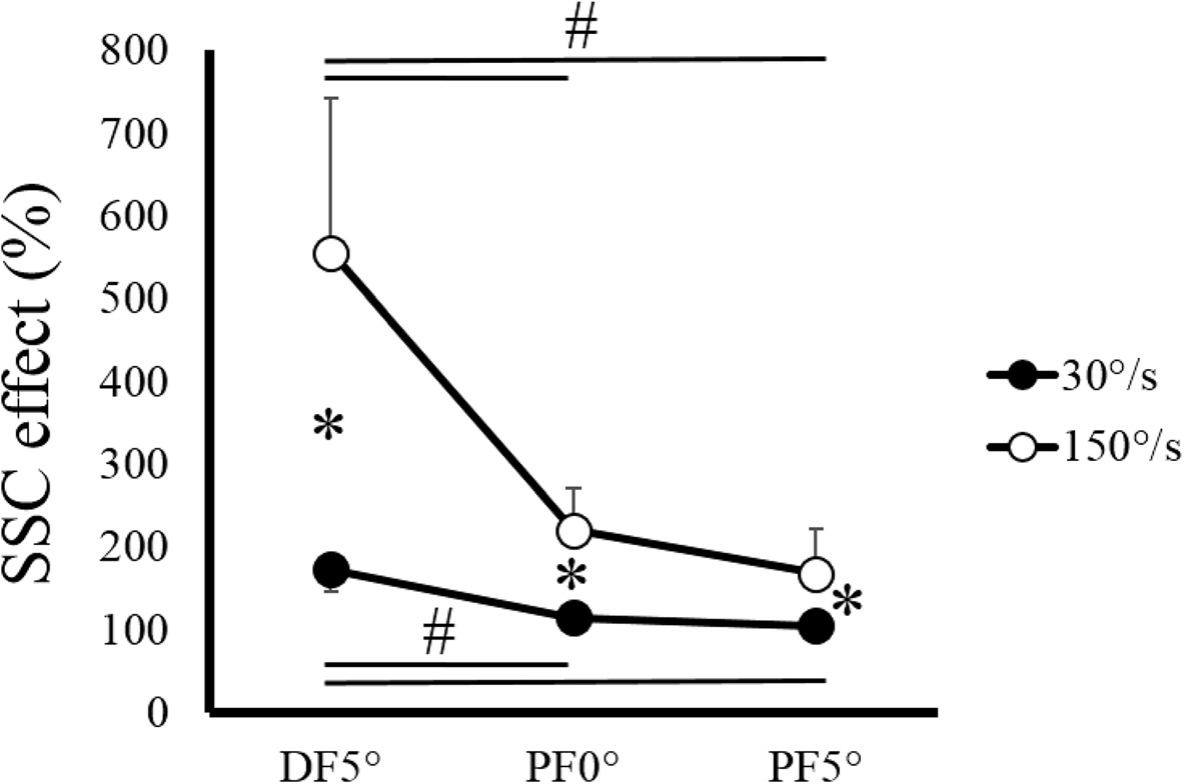
**Stretch-shortening cycle (SSC) effect. SSC effect between 30°/s (filled circle) and 150°/s (open circle) conditions recorded at each joint angle.** # indicates a significant difference among joint angles. *indicates a significant difference between joint angular velocities. Abbreviations: DF, dorsiflexion; PF, plantarflexion.

Two-way ANOVA revealed a significant interaction (*F* = 54.345, partial *η*^2^ = 0.832, *p* < 0.001). Subsequent one-way ANOVA and post hoc test showed that SSC effect was significantly larger in the 150°/s condition than in the 30°/s condition (DF5°, *p* < 0.001; PF0°, *p* < 0.001; PF5°, *p* = 0.001) (Figure 1). Moreover, in both velocity conditions, SSC effect was significantly larger at DF5° than at PF0° and PF5° (*p* < 0.001), whereas SSC effect was not significantly different between PF0° and PF5° (150°/s condition, *p* = 0.456; 30°/s condition, *p* = 0.563).

### Discussion

The purpose of this study was to examine the influence of joint angular velocity on SSC effect. The results demonstrated that the magnitude of SSC effect was larger when joint angular velocity was larger. In addition, the magnitude of SSC effect was larger in the early phase of contraction (i.e., at DF5° compared with at PF0° and PF5°).

Our current results are similar to those of a previous study (Svantesson et al. 1994), which found that SSC effect was larger in a higher joint angular velocity condition. In the current study, since neural activity remained constant (i.e., identical stimulation voltage) we can say that velocity-dependence of SSC effect is not caused by neural activity.

We suggest that this phenomenon can be explained by preactivation which is one of the mechanisms of SSC effect (Bobbert et al. 1996; Ettema et al. 1990). Without prior contraction, it takes several seconds to reach maximal force (or torque) (Andersen and Aagaard 2006; Aagaard et al. 2002); in other words, activation level is still submaximal in the early phase of contraction. On the other hand, with prior contraction, the delay to increase activation level of the muscle can be avoided (Gransberg and Knutsson 1983; Jensen et al. 1991). Thus, activation level is high, even in the early phase of contraction. This preactivation effect is substantial when the time to increase activation level is short. Considering the fact that at a given range of motion, duration of contraction decreases as the angular velocity increases, the influence of preactivation on SSC effect must be large when joint angular velocity is large.

Our result that the magnitude of SSC effect was larger in the early phase of contraction also supports the idea that preactivation has a substantial influence on SSC effect. Specifically, it is obvious that activation level is lower in the early phase than in the latter phase of contraction. Therefore, the effect of avoiding the delay to increase activation level (that is, preactivation) has a larger influence on the early phase of contraction than on the latter phase of contraction. Based on these findings, using the counter-movement (i.e., stretch-shortening cycle) is important for improving performance especially in the motions that require large velocities because the duration for increasing activation level is limited in these motions.

There are other factors that can affect SSC effect, including stretch reflex (Dietz et al. 1979; Nichols and Houk 1973), tendon elongation (Finni et al. 2003; Kawakami et al. 2002), and residual force enhancement (Edman et al. 1982; Joumaa et al. 2008). First, since the current study adopted electrically evoked contractions, the influence of stretch reflex is discarded. Second, the other two factors, tendon elongation and residual force enhancement also would not explain the observed difference in SSC effect between the two velocity conditions. Considering the facts that these two mechanisms are caused by the elongation of muscle-tendon complex (Edman et al. 1982; Kawakami et al. 2002) and that the range of motion and velocity of eccentric contraction (i.e., elongation of muscle-tendon complex) were identical between 30°/s and 150°/s conditions, influence of the tendon elongation and residual force enhancement should be included to a similar extent in both velocity conditions. Therefore, the observed difference in SSC effect would not be caused by tendon elongation or residual force enhancement.

### Conclusion

We confirmed that SSC effect was large when joint angular velocity was large. Because the current study adopted electrically-evoked contraction, observed velocity-dependence of SSC effect was not caused by neural activity. We speculate that this relationship would be caused by the preactivation. When joint angular velocity is large, time to increase activation level is limited. The effect of preactivation (one of the mechanisms of SSC effect) is substantial when time to increase activation level is limited.
